# Design parameters for enhanced photon absorption in vertically aligned silicon nanowire arrays

**DOI:** 10.1186/1556-276X-9-511

**Published:** 2014-09-19

**Authors:** Stefan T Jäger, Steffen Strehle

**Affiliations:** 1 Institute of Electron Devices and Circuits, Ulm University, Albert-Einstein-Allee 45, 89081 Ulm, Germany

**Keywords:** Nanowire array, Solar cell, Photon harvesting, Light absorption, 3D simulations

## Abstract

Superior photon absorption in ordered nanowire arrays has been demonstrated recently. However, systematic studies are still missing to explore the limits of their implementation as functional photonic devices. With emphasis on silicon nanowires, we investigated the effects of nanowire diameter, length, morphology, and pitch on the photon absorption within the visible solar spectrum based on simulations. Our results reveal that these parameters are crucial but disclose a path to improve the absorbance drastically.

**PACS:**

78.40.Fy; 78.67.Uh; 78.67.-n

## Background

**Figure 1:**
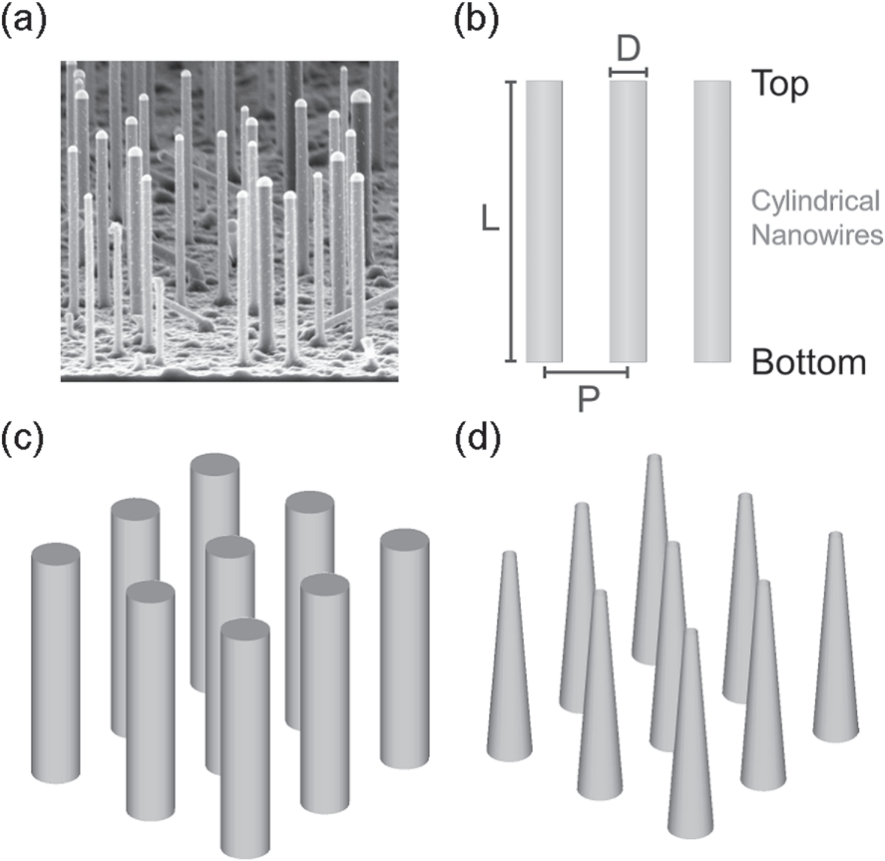
**SEM image of a nanowire array, illustration of geometry parameters, and nanowire morphology schematics.(a)** SEM image of a typical SiNW nanowire array synthesized bottom-up by gold catalyzed VLS. **(b)** 2D illustration of the geometric parameters: diameter D, length L, and pitch P of cylindrical NWs. **(c)** Schematic illustration of cylindrical and **(d)** tapered NWs. All simulations were conducted three dimensionally without an additional substrate.

Photon harvesting is one key factor determining the performance of photonic devices such as solar cells and photodetectors. In recent years, promising improvements could be accomplished for photon management utilizing nanostructures such as wires, cones, dots, pyramids, and cubes to lower the reflectivity [1], increase the absorption over broad wavelength ranges [2], and to reduce the dependency on the angle of light incidence [2-4]. Despite these achievements, further systematic studies are required to enable rational tailoring of nanoscale morphologies with respect to the optical application. Within the range of potential nanostructures, nanowires (NW) are one important class [5-9]. The focus of this work is put on silicon nanowires (SiNWs), which have been in discussion for a while as effective structures to drastically improve the photon absorption probability especially if aligned in parallel to the angle of light incidence [10,11]. Aside from this passive exploitation of the geometry, SiNWs can possess a p-n junction as well allowing their direct use for solar energy conversion if assembled as photonic device [12-17]. In terms of the material, silicon is still leading for semiconductor photovoltaic applications but is also vastly used in various microfabricated electronic devices enabling perfect alignment of silicon nanostructures with common standards in technology. Aligned SiNW arrays can be fabricated bottom-up, e.g., by the vapor-liquid-solid method (VLS, Figure 1a) [18] or by top-down etching techniques [19]. Although both methods have their intrinsic advantages and disadvantages, they have in common that with respect to the geometry, the average nanowire pitch, length, diameter, and overall morphology such as nanowire tapering can in principle be rationally adjusted. As shown in individual experiments, these parameters are key factors for the resulting optical properties [20,21]. For instance, Zhu et al. showed that tapered SiNWs excel over cylindrical nanowires in terms of a minimized reflectivity [22]. To explore the limits of silicon nanowire arrays utilized for photonic applications, we performed comprehensive three-dimensional (3D) simulations for idealized vertical SiNW arrays in comparison to the literature and mainly thin single crystalline silicon membranes. From the simulations, a general set of design parameters can be deduced, enabling to pre-evaluate the optical properties depending on the geometrical conditions preventing intrinsic design-based deficiencies of devices within experiments.

## Methods

**Figure 2:**
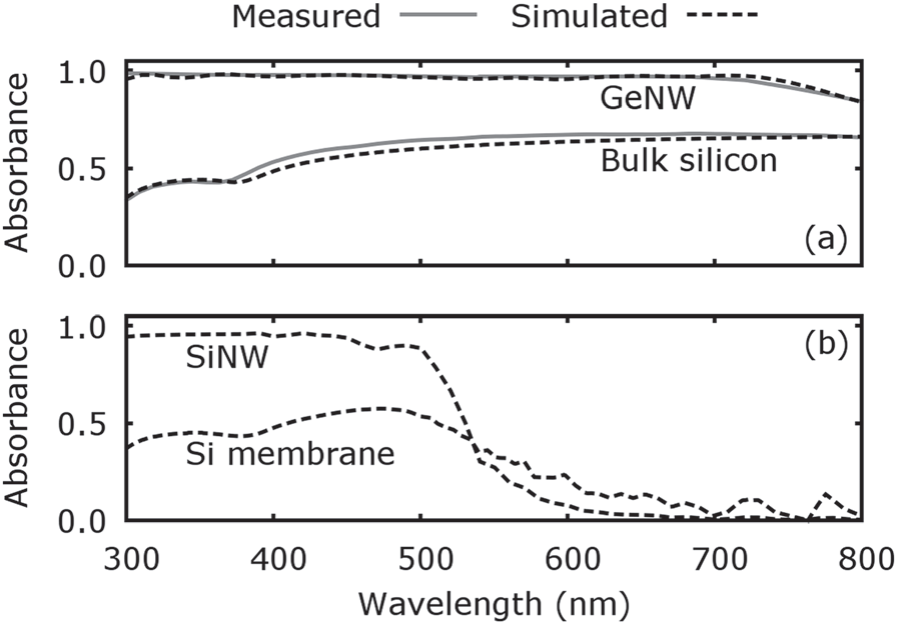
**Measured absorption spectra for bulk silicon and GeNW arrays and simulated spectral absorbance.(a)** Absorption spectra for bulk silicon and a GeNW array with NWs of 2 µm in length, 80 nm in diameter and 150 nm pitch situated on a glass substrate. The measured GeNW spectrum was extracted from Fan et al. [25]. The spectra were measured and simulated with a homogenous, non-wavelength-dependent illumination and are therefore not weighted with an AM1.5G spectrum. **(b)** Simulated spectral absorbance for a substrate-free SiNW array equal in its arrangement to the aforementioned GeNW array and in comparison to a Si membrane of 2 µm in thickness.

The 3D optical simulations were realized by means of the finite element method (FEM). FEM simulations are very suitable to simulate NW arrays with various shapes as they use a variable mesh to model the desired geometry. In principle, two NW shapes were studied: NWs with a constant diameter D (cylindrical NWs, Figure 1b,c) and tapered NWs with a linear transition from a fixed bottom diameter to a smaller top diameter (Figure 1d). To describe wavelength-dependent light-matter interaction, all simulations were conducted in the frequency domain and were based on Maxwell’s equations but without considering thermal effects. Although the infrared wavelength regime is important, the following studies focus on the spectral range from 300 to 800 nm. This is based on the fact that SiNW arrays and thin Si membranes do not show any significant absorption above 800 nm in contrary to bulk silicon [see Additional file 1: Figure S1] [20]. For all considered scenarios, the simulated sample comprised of a homogenous infinite set of nanowires placed in air with a specific NW length and periodic pitch (Figure 1b). The infinite NW array eliminates the influence of any boundary effects and is created by periodic repetition of a single nanowire element in two lateral directions. Consequently, all results can be ascribed to geometric and material effects solely. Material parameters were implemented in our simulations as wavelength-dependent complex refractive indices [see Additional file 1: Figure S1]. The optical illumination spectrum was realized with electromagnetic waves propagating as plane waves perpendicular to the surface and parallel to the NWs longitudinal axis and was, if not stated otherwise, weighted with an air-mass 1.5 global (AM 1.5G) solar spectrum [23]. The optical transmittance and reflectance were directly extracted from the simulations depending on the wavelength in the aforementioned range of 300 to 800 nm with an increment of 10 nm. For comparison purposes, the ideal short circuit current density *J*_SC_ was calclulated from the obtained absorption spectrum considering an internal quantum efficiency of 0.8 (approximately mean value from [24]) and 1 sun illumination. With respect to maximum accuracy, the FEM mesh size was chosen to be much smaller than the simulated wavelengths with a varying edge length between 14 and 30 nm. Finally, the absorbance was determined from the simple relation that the summation of transmittance, reflectance, and absorbance equals one. All simulations were conducted under similar conditions. Therefore, the calculated short circuit current density leads to a numeric value suitable to quantitatively compare the presented simulations among each other and even with experiments (Figure 2a). Our simulations were performed without a substrate in order to keep the obtained results as generic as possible with emphasis on nanowires and unaffected from a substrate material (e.g., additional reflection or absorption). Hence, the nanowire array is basically floating in air enabling us to evaluate its optical behavior solely. This approach is supported by van de Groep and Polman [21], who showed that a substrate material is mainly influencing the absolute values while the overall characteristics and trends are governed by the used nanostructures.

## Results and discussion

To validate our simulation approach experimentally, a single crystalline (100) silicon wafer was spectroscopically characterized, allowing us to extract the absorbance depending on the wavelength. In Figure 2a, these values are compared to the results deduced from our simulation considering a planar structure with infinite thickness. The results show that our simulation can appropriately predict the optical behavior of a real bulk silicon sample. Optical characterizations performed by other groups with SiNW arrays are also qualitatively in suitable agreement with our results [20]. However, a quantitative comparison would require samples with well-defined structures over large scales and the full experimental parameter set if published elsewhere. While until now, these constraints appear unfulfilled for SiNW arrays; results published by Fan et al. [25] for germanium (Ge) NW arrays appear adequate. In this paper, cylindrical NWs with 2 µm in length and 80 nm in diameter, both average values, are arranged with an average pitch of 150 nm on a glass substrate. Taking the ideal character of the simulations, the tabulated optical properties, and the experimental conditions into account, the simulation is in convincing agreement (Figure 2a).

A first pre-evaluation of a substrate-free SiNW array, equal to the aforementioned GeNW array, is shown in comparison to a planar Si membrane in Figure 2b. To enable a comparison, the membrane thickness and the NW length were both fixed to 2 µm. These simulations indicate that the SiNW array exhibits a significantly increased absorption (approximately factor 1.4) over a large spectral range. This is even more supported by the fact that in this case, the NW array volume is less then 23% of the membrane material volume. A straightforward explanation is the higher bulk surface reflectivity. In the visible wavelength range, a mean ratio of about 50% of incident light is reflected in case of silicon and is therefore not coupled into the material. In contrast, NW arrays and the surrounding air are minimizing reflective losses by creating an effective refractive index. Its value or gradient depends intrinsically on the NW geometry and arrangement which is also pointed out, e.g., by Patchett et al. [26], which even enables us to rationally customize spectral absorption characteristics [see Additional file 1: Figure S2]. However, the focus of this study is to maximize the absorption or short circuit current density in NW arrays depending on the parameters NW diameter, morphology, length, and pitch. If the spectral characteristics of the silicon examples given in Figure 2a,b are compared to previous works, it can be deduced that the longer wavelength regime is suppressed as a result of the distinct material volume and thickness (NW length) dependency of the optical absorption [20].

**Figure 3:**
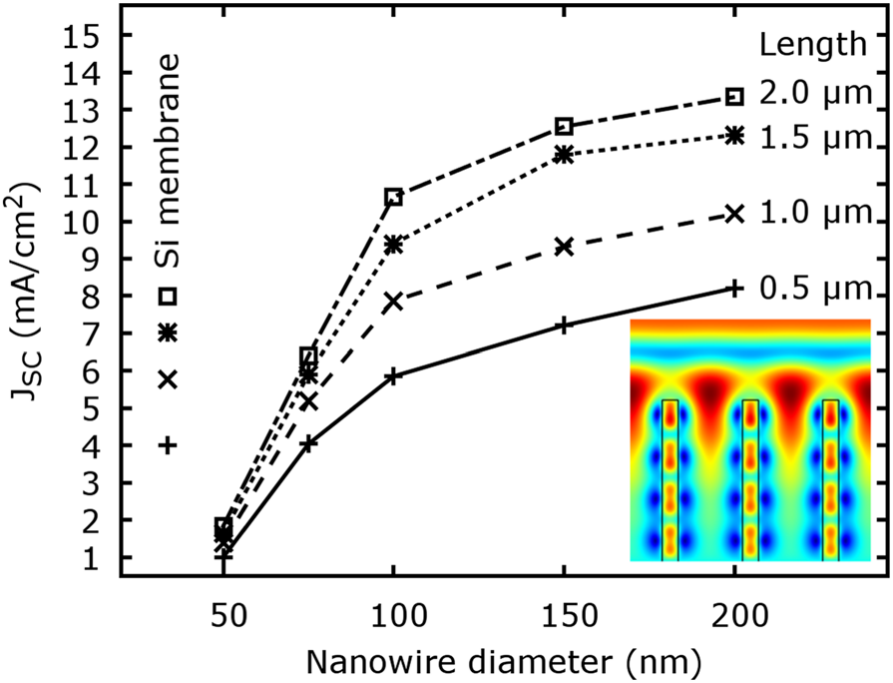
**Short circuit current density characteristics depending on NW diameter (cylindrical shape) and length.** At 250 nm constant pitch showing the tradeoff between NW surface reflection and material volume in comparison to silicon membrane material. Inset: exemplary electric field distribution inside a NW array for light of 800 nm wavelength (blue represents the lowest and red the highest electric field). Short circuit current density values are plotted for corresponding Si membrane thicknesses.

**Figure 4:**
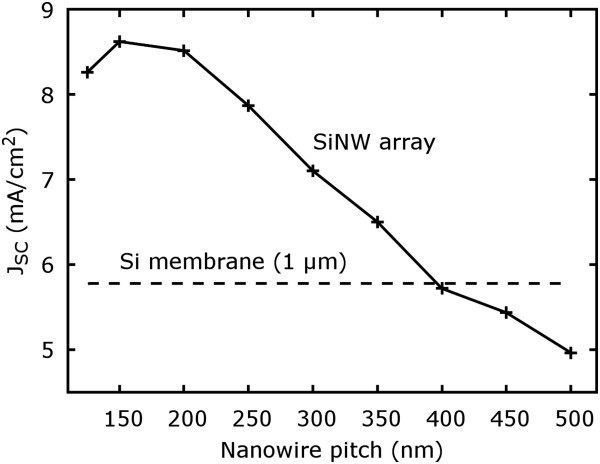
**Short circuit current density for the variation of the pitch in the range of 125 to 500 nm (NW diameter 100 nm, length 1 µm).** The maximum represents the optimum of increased material volume with smaller pitch and minimized surface reflection with a larger pitch.

To evaluate material volume effects, the NW diameter and length were varied from 50 to 200 nm and from 0.5 to 2 µm, respectively, using a constant pitch of 250 nm (Figure 3). Figure 3 shows that the short circuit current density increases with increasing length and diameter (total absorption is provided in Additional file 1: Figure S5 to S8). However, the gain in current density is not proportional to the gain in material volume. For instance, only moderate current density improvements can be achieved for diameters larger than 100 nm, which stands in striking contrast to the strong increase for smaller diameters. Depending on the diameter, but also pitch, the silicon/air volume ratio varies strongly. For instance, a diameter of 150 nm and a pitch of 250 nm make up for a volume filling of silicon of about 30%. However, a short circuit current density enhancement of about 70% is achieved. Here, mainly two effects contribute to these results. The effect of the diameter can be simply understood by considering the aforementioned effective refractive index approximation [26]. On the one hand, the material volume and therefore, the short circuit current density, is increased with increasing diameter. On the other hand, the reflection coefficient is increased as well based on the fact that the structure is continuously approaching its bulk state [1]. In case of the length, the absorption is dominated by the upper part of the NW (Figure 3 inset). Therefore, the gain in absorption and short circuit current density declines non-linearly with increasing length. From the simulations, a representable absorption coefficient based on 100 nm NW diameter and 250 nm pitch for 500 nm wavelength of 1.3×10^6^ 1/m could be extracted. This means that 90% of the incident light is already absorbed within the first 1.8 µm in this case.The pitch, which was kept constant at 250 nm before, should now be evaluated exemplary for a NW length of 1 µm and a diameter of 100 nm within a range of 125 to 500 nm referring to the highest and lowest NW density, respectively. As shown in Figure 4, the short circuit current density has a maximum in the range of 150 to 200 nm pitch and decreases to both sides. Above 200 nm, the current density declines almost linearly until it approaches the short circuit current density for planar membrane silicon.

**Figure 5:**
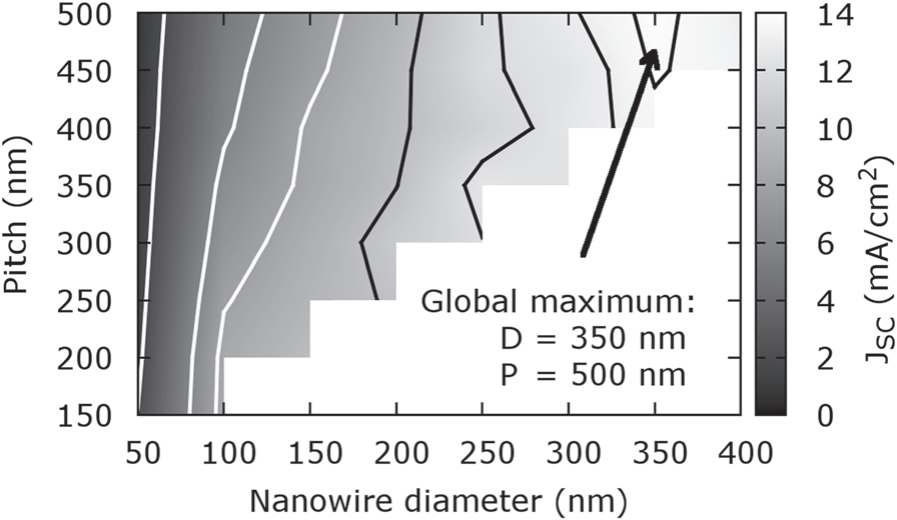
**Short circuit current density for NW arrays with different NW pitch and diameter.** The length was fixed to 1 µm. Contour lines symbolize parameter combinations with equal short circuit current density. A global maximum is achieved for NW arrays with approximately 350 nm NW diameter and 500 nm pitch.

Based on the discussed fact that the absolute value of the short circuit current density depends strongly on the pitch and diameter, both must also be evaluated simultaneously in order to determine the global maximum in photon absorption or current density, as shown in Figure 5. This maximum is shifted towards the micron scale and can be found at a pitch of 500 nm using cylindrical wires of 350 nm in diameter. For these parameters, the maximum short circuit current density is obtained with a value of 13.9 mA/cm ^2^, which is over a factor of two higher than 5.8 mA/cm ^2^ obtained for a silicon membrane of 1 µm in thickness. In general, a characteristic local maximum can be expected if the D/P ratio is in the range of 0.7 to 0.8, which is in alignment with estimates published elsewhere [27-29].

**Figure 6:**
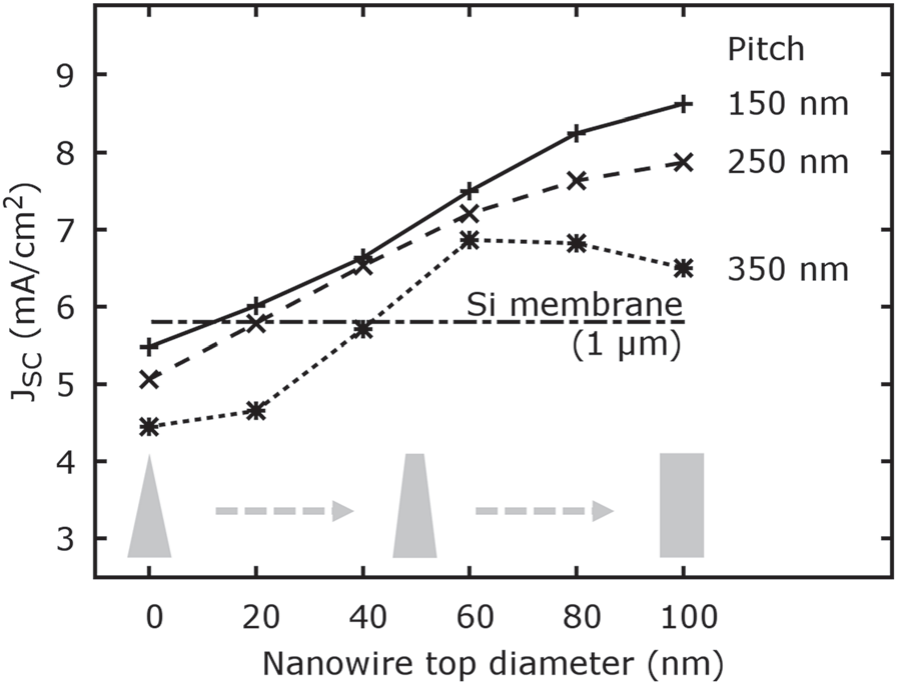
**Short circuit current density of NW arrays with varying pitch from 150 to 350 nm depending on the degree of tapering.** The NW length and the bottom diameter were fixed to 1 µm and 100 nm, respectively.

Until now, only SiNWs with cylindrical geometry were considered in the discussion. However, in reality, the axial geometry can easily differ from this model being more conical or frustum like (Figure 1d). Such tapered structures are, for instance, readily obtained for VLS synthesis if a lateral overgrowth is present besides the axial NW elongation. In principle, tapered NW structures possess an axial linear diameter gradient providing simultaneously an axial gradient for the effective refractive index. However, a thorough discussion of tapering effects is complex and depends strongly on the aspired application. To be in alignment with the previous simulations, NWs with 100 nm in diameter are considered exemplarily as depicted in Figure 6. Therefore, NW arrays were studied with a fixed NW bottom diameter of 100 nm while the top diameter was varied from 0 nm (ideal sharp tip) to 100 nm (cylindrical NW). Any structure in between is consequently a truncated cone. As shown in Figure 6, strong tapering with a top diameter of less then 60 nm does not provide a benefit in terms of a maximized photon absorption or short circuit current density. Nevertheless, moderate tapering or truncated cones can represent an ideal geometry (see 350 nm pitch in Figure 6). This is as well valid for the previously derived global maximum with 350 nm NW diameter at 500 nm pitch. If this structure is turned into a truncated cone with 250 nm top diameter, the short circuit current density can be further increased by 9.4% to 15.2 mA/cm ^2^. In general, the achievable enhancement or the ideal geometry depends strongly on all geometric factors like diameter, length, and pitch influencing again the tradeoff between volume and effective index of refraction.

A NW shape tending towards a cylinder should in general perform better than highly tapered nanowires. This is also still in agreement with the reported low reflectance values for strongly tapered nanowires situated on a substrate [1,30]. Tapered NW arrangements benefit from an enhanced anti-reflection effect based on the tunable gradient of the effective refractive index from air to bulk material. This allows to maximize the transmittance or to minimize substrate reflectance if required for the application [see Additional file 1: Figure S3 and Additional file 1: Figure S4].

## Conclusions

In conclusion, we evaluated the absorption characteristics of ordered arrays of vertically aligned silicon nanowires depending on their diameter, pitch, length, and shape using comprehensive 3D FEM simulations. From the simulations, the short circuit current density was derived, and we showed that silicon NW arrays can surpass the absorbance of silicon membranes. The global maximum of the short circuit current density for cylindrically shaped NWs could be predicted for approx. 350 nm diameter with 500 nm pitch with a short circuit current density of 13.9 mA/cm ^2^. This value can be further increased to 15.2 mA/cm ^2^ if the NWs represent a truncated cone with 250 nm top diameter. However, the implementation of highly tapered nanowires cannot be recommended in general if a maximum short circuit current density should be achieved within the NW array at minimum material volume.

## Abbreviations

D: nanowire diameter; FEM: finite element method; Ge: germanium; GeNW: germanium nanowire; J _SC_: short circuit current density; L: nanowire length; NW: nanowire; P: nanowire pitch; Si: silicon; SiNW: silicon nanowire; VLS: vapor-liquid-solid.

## Competing interests

The authors declare that they have no competing interests.

## Authors’ contributions

SJ designed the studies, carried out the simulations, did the experiments, and drafted the manuscript. SS conceived the studies, advised on the entire project, and finalized the manuscript. Both authors read and approved the final manuscript.

## Supplementary Material

Additional file 1 **Figures S1 to S9.Figure S1.** Wavelength-dependent complex refractive index of silicon utilized for our simulations. **Figure S2.** Absorption spectra for NW arrays with different NW diameters at 250 nm pitch and 1 µm length. Rational tailoring of the absorption characteristics can be achieved by NW geometry variation. **Figure S3.** Total reflection of NW arrays with varying pitch from 150 to 350 nm depending on the degree of tapering. The NW length and the bottom diameter were fixed to 1 µm to 100 nm, respectively. **Figure S4.** Total transmission of NW arrays with varying pitch from 150 to 350 nm depending on the degree of tapering. The NW length and the bottom diameter were fixed to 1 µm to 100 nm, respectively. **Figure S5.** Absorption characteristics depending on NW diameter (cylindrical shape) and length at 250 nm constant pitch showing the tradeoff between NW surface reflection and material volume in comparison to silicon membrane material. Inset: exemplary electric field distribution inside a NW array for light of 800 nm wavelength (blue represents the lowest and red the highest electric field). Total absorption values are ploted for corresponding Si membrane thicknesses. **Figure S6.** Total absorption for the variation of the pitch in the range of 125 to 500 nm (NW diameter 100 nm, length 1 µm). The maximum represents the optimum of increased material volume with smaller pitch and minimized surface reflection with a larger pitch. Ideal short circuit current densities are displayed for the maximum total absorption at 150 nm NW pitch and the Si membrane. **Figure S7.** Total absorption for NW arrays with different NW pitch and diameter. The length was fixed to 1 µm. Contour lines symbolize parameter combinations with equal total absorption. A global maximum is achieved for NW arrays with approximately 350 nm NW diameter and 500 nm pitch. **Figure S8.** Total absorption for NW arrays with varying pitch from 150 to 350 nm depending on the degree of tapering. The NW length and the bottom diameter were fixed to 1 µm and 100 nm, respectively. **Figure S9.** Short circuit current densities for NW arrays with optimal parameters (350 nm diameter and 500 nm pitch). NW length is 1 µm. Click here for file
