# Supplementary material for: Design parameters for enhanced photon absorption in vertically aligned silicon nanowire arrays
Source: Nanoscale Res Lett. 2014 Sep 19;9(1):511. doi: 10.1186/1556-276X-9-511 (PMC4177724; doi:10.1186/1556-276X-9-511)
Supplement: Additional file 1 — Figures S1 to S9.Figure S1. Wavelength-dependent complex refractive index of silicon utilized for our simulations. Figure S2. Absorption spectra for NW arrays with different NW diameters at 250 nm pitch and 1 µm length. Rational tailoring of the absorption characteristics can be achieved by NW geometry variation. Figure S3. Total reflection of NW arrays with varying pitch from 150 to 350 nm depending on the degree of tapering. The NW length and the bottom diameter were fixed to 1 µm to 100 nm, respectively. Figure S4. Total transmission of NW arrays with varying pitch from 150 to 350 nm depending on the degree of tapering. The NW length and the bottom diameter were fixed to 1 µm to 100 nm, respectively. Figure S5. Absorption characteristics depending on NW diameter (cylindrical shape) and length at 250 nm constant pitch showing the tradeoff between NW surface reflection and material volume in comparison to silicon membrane material. Inset: exemplary electric field distribution inside a NW array for light of 800 nm wavelength (blue represents the lowest and red the highest electric field). Total absorption values are ploted for corresponding Si membrane thicknesses. Figure S6. Total absorption for the variation of the pitch in the range of 125 to 500 nm (NW diameter 100 nm, length 1 µm). The maximum represents the optimum of increased material volume with smaller pitch and minimized surface reflection with a larger pitch. Ideal short circuit current densities are displayed for the maximum total absorption at 150 nm NW pitch and the Si membrane. Figure S7. Total absorption for NW arrays with different NW pitch and diameter. The length was fixed to 1 µm. Contour lines symbolize parameter combinations with equal total absorption. A global maximum is achieved for NW arrays with approximately 350 nm NW diameter and 500 nm pitch. Figure S8. Total absorption for NW arrays with varying pitch from 150 to 350 nm depending on the degree of tapering. The NW length and the bottom [file 1556-276X-9-511-S1.pdf]

Design parameters for enhanced photon absorption in vertically aligned silicon nanowire arrays

Stefan T. Jäger and Steffen Strehle

Additional file 1 - Supplementary figures S1, S2, S3, S4, S5, S6, S7, S8, and S9

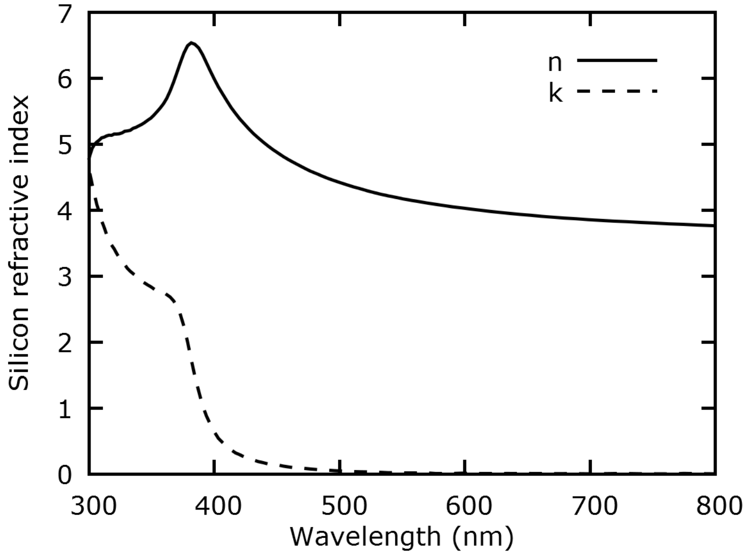

Figure S1 Wavelength dependent complex refractive index of silicon utilized for our simulations. Taken from Palik, E.D.: Handbook of Optical Constants of Solids, (1991).

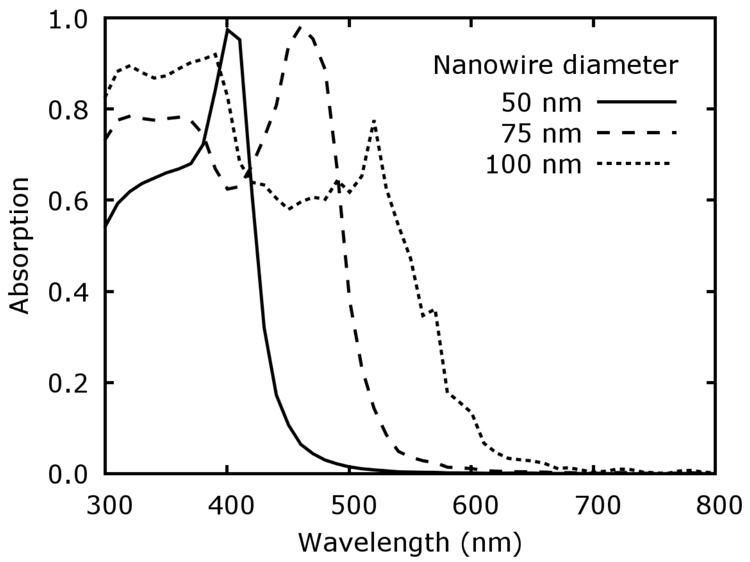

Figure S2 Absorption spectra for NW arrays with different NW diameters at 250 nm pitch and 1 µm length. Rational tailoring of the absorption characteristics can be achieved by NW geometry variation.

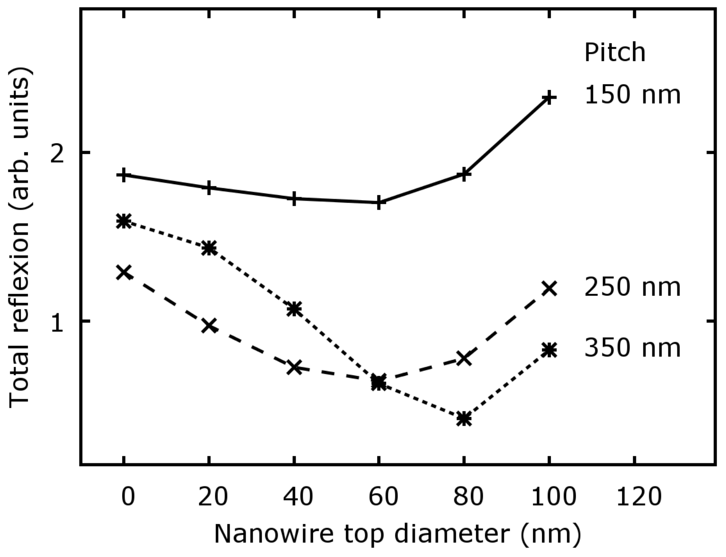

Figure S3 Total reflexion of NW arrays with varying pitch from 150 to 350 nm in dependence on the degree of tapering. The NW length and the bottom diameter were fixed to 1 µm to 100 nm, respectively.

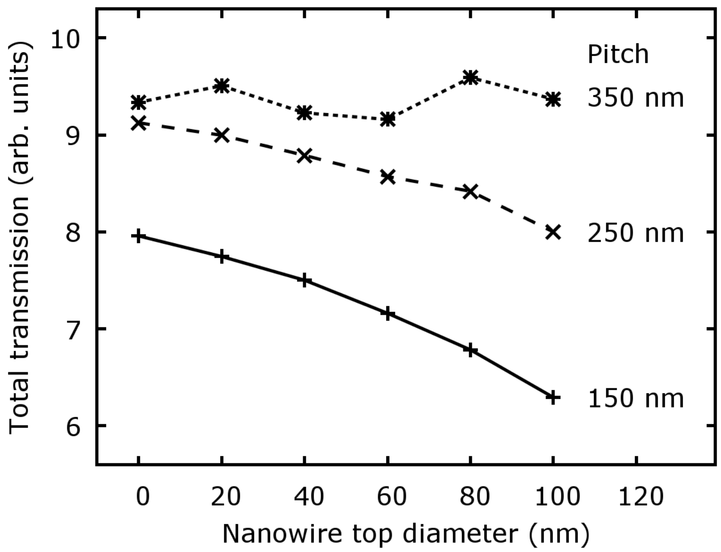

Figure S4 Total transmission of NW arrays with varying pitch from 150 to 350 nm in dependence on the degree of tapering. The NW length and the bottom diameter were fixed to 1 µm to 100 nm, respectively.

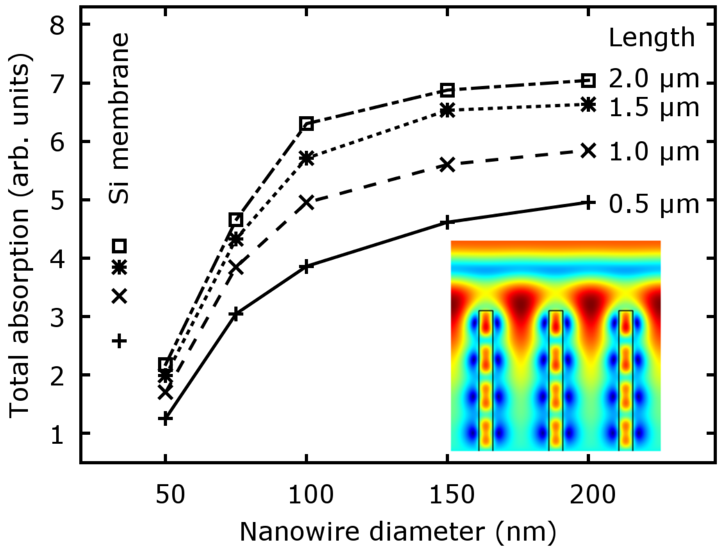

Figure S5 Absorption characteristics in dependence on NW diameter (cylindrical shape) and length at 250 nm constant pitch showing the tradeoff between NW surface reflection and material volume in comparison to silicon membrane material. Inset: exemplary electric field distribution inside a NW array for light of 800 nm wavelength (blue represents the lowest and red the highest electric field). Total absorption values are ploted for corresponding Si membrane thicknesses.

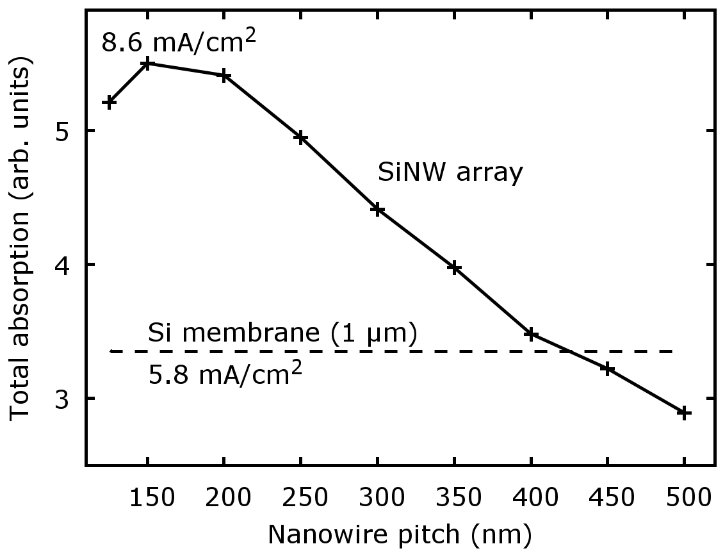

Figure S6 Total absorption for the variation of the pitch in the range of 125 to 500 nm (NW diameter 100 nm, length 1 μm). The maximum represents the optimum of increased material volume with smaller pitch and minimized surface reflection with a larger pitch. Ideal short circuit current densities are displayed for the maximum total absorption at 150 nm NW pitch and the Si membrane.

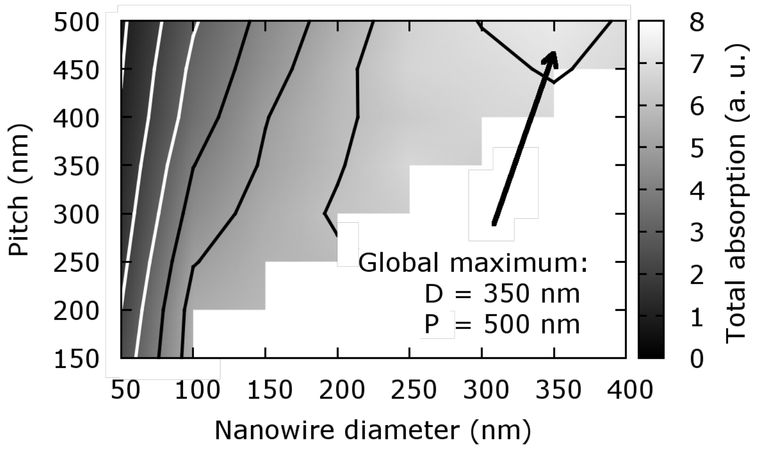

Figure S7 Total absorption for NW arrays with different NW pitch and diameter. The length was fixed to 1 μm. Contour lines symbolize parameter combinations with equal total absorption. A global maximum is achieved for NW arrays with approx. 350 nm NW diameter and 500 nm pitch.

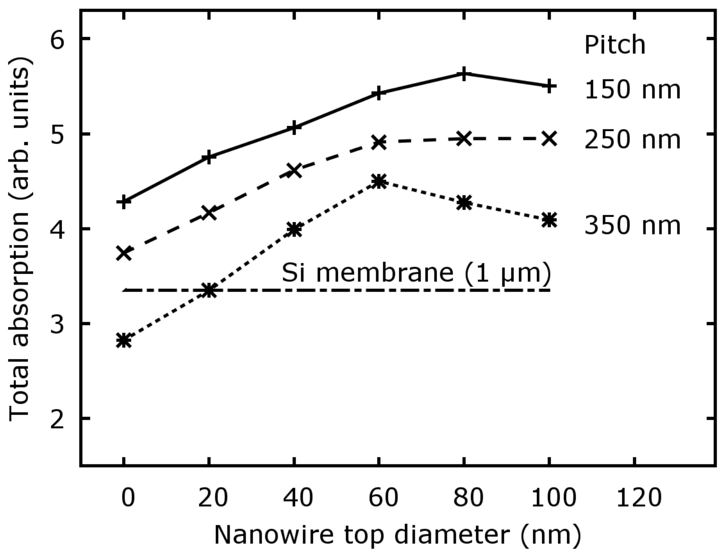

Figure S8 Total absorption for NW arrays with varying pitch from 150 to 350 nm in dependence on the degree of tapering. The NW length and the bottom diameter were fixed to 1 µm and 100 nm, respectively.

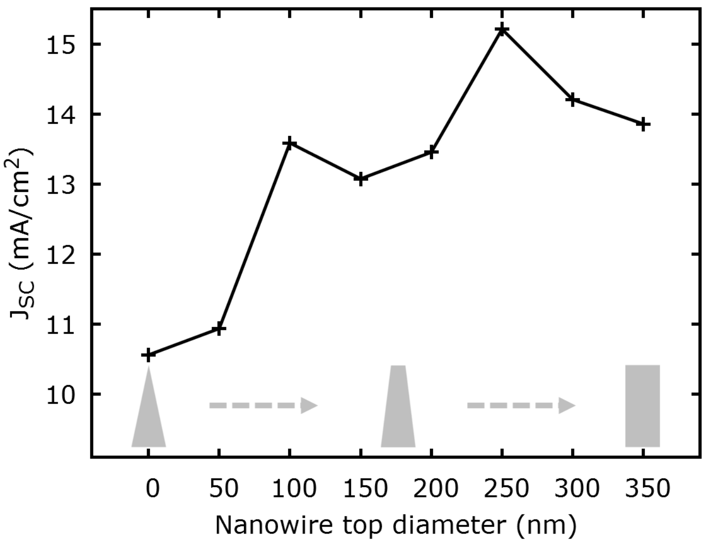

Figure S9 Short circuit current densities for NW arrays with optimal parameters (350 nm diameter and 500 nm pitch). NW length is 1 µm.
